# Computational insights on asymmetrical $D_{1}$ and $D_{2}$ receptor-mediated chunking: implications for OCD and Schizophrenia

**DOI:** 10.1007/s11571-022-09865-4

**Published:** 2023-01-12

**Authors:** Krisztina Szalisznyó, David N. Silverstein

**Affiliations:** 1 Department of Medical Sciences, Psychiatry, Uppsala University Hospital, Uppsala University, 751 85 Uppsala, Sweden; 2 Theoretical Neuroscience and Complex Systems Research Group, Wigner Research Centre for Physics, Budapest, Hungary; 3 Agora for Biosystems, Sigtuna Foundation, Sigtuna, Sweden

**Keywords:** Chunking, Reservoir computing, Dopamine receptors, Striatum

## Abstract

Repetitive thoughts and motor programs including perseveration are bridge symptoms characteristic of obsessive compulsive disorder (OCD), schizophrenia and in the co-morbid overlap of these conditions. The above pathologies are sensitive to altered activation and kinetics of dopamine $D_{1}$ and $D_{2}$ receptors that differently influence sequence learning and recall. Recognizing start and stop elements of motor and cognitive behaviors has crucial importance. During chunking, frequent components of temporal strings are concatenated into single units. We extended a published computational model (Asabuki et al. 2018), where two populations of neurons are connected and simulated in a reservoir computing framework. These neural pools were adopted to represent D_1_ and D_2_ striatal neuronal populations. We investigated how specific neural and striatal circuit parameters can influence start/stop signaling and found that asymmetric intra-network connection probabilities, synaptic weights and differential time constants may contribute to signaling of start/stop elements within learned sequences. Asymmetric coupling between the striatal $D_{1}$ and $D_{2}$ neural populations was also demonstrated to be beneficial. Our modeling results predict that dynamical differences between the two dopaminergic striatal populations and the interaction between them may play complementary roles in chunk boundary signaling. Start and stop dichotomies can arise from the larger circuit dynamics as well, since neural and intra-striatal connections only partially support a clear division of labor.

## Introduction

The basal ganglia and striatum are critically involved in sensorimotor chunking, constructing performance units of sequence representations that once learned, can be treated as separate entities (Graybiel 1998; Solopchuk et al. 2016; Jin and Costa 2010; Jin et al. 2014). What are the computational advantages of the two parallel dopaminergic receptor systems ($D_{1}$ and $D_{2}$ receptor families) in chunk learning and recall? The classical view is that stimulation of striatal dopamine $D_{1}$ receptors of GABA-ergic principal medium spiny neurons (MSN) facilitates the direct pathway activity and movement, while dopamine $D_{2}$ receptors of MSNs influences the indirect pathway and inhibits movement or competing actions (Gerfen and Surmeier 2011; Cruz et al. 2022; Cui et al. 2013). Imbalances in the activity of the $D_{1}$-expressing direct pathway and $D_{2}$-expressing indirect pathway MSNs can contribute to basal ganglia disorders (Krajeski et al. 2019).

Recurrent collateral connections among MSNs are not symmetrical. $D_{2}$ MSNs have additional and stronger inhibitory connections on $D_{1}$ MSNs than vice versa (Planert et al. 2010; Taverna et al. 2008). Thus, the dominating interaction between these two projection systems relies mostly on collateral projections from $D_{2}$ MSNs to $D_{1}$ MSNs.

Phasic dopamine release primarily increases $D_{1}$ occupancy, whereas $D_{2}$ occupancy is less affected (Dreyer et al. 2010). Low-level baseline tonic dopamine release is sufficient for altering $D_{2}$ receptors (Schultz 2007; Durstewitz and Seamans 2008). Dopamine can bias action selection by modulating striatal direct and indirect pathways (Howard et al. 2017).

Detection and marking of chunk boundaries over time and adjusting the boundary detection threshold is a dynamic and crucial process for adaptive habit formation (Ramkumar et al. 2016; Barnes et al. 2005). Sequence segmentation can rely on detecting boundaries between events by transients (Asabuki et al. 2018). It is likely that different mechanisms with increasing degrees of abstraction representing sequence knowledge can operate in parallel to each other and both chunking as well as transitioning might benefit from recognizing start / stop signals (Dehaene et al. 2015).

### Dopamine in excessively repetitive behavior in animals

OCD can involve highly complex stereotypies of action and thought sequences (Taylor 2010). A rodent study found that dopamine agonists and both $D_{1}$ and $D_{2}$ receptors can modulate the duration of OCD behavior (Hoffman 2012). A $D_{2}$ agonist increased the total duration and frequency of compulsive checking behavior (Hoffman 2012). The excessive repetition of grooming sequences observed in the $D_{1}$ agonist-treated rat has been speculated to be a potential model for complex tics observed in OCD and Tourette syndrome (Taylor 2010). Concurrent stimulation of $D_{1}$ and $D_{2}$ receptors in the dorsal caudate-putamen enhanced both locomotor activity and stereotypy.

Rodent studies show that there are cycles of behavior, which are relatively indivisible rigid behavioral units or “chunks”. The decision of whether or not to enter a new behavioral cycle is an important control point and could depend on ${D}_{1}$ receptor dynamics. In rats, pharmacological boosting by dopamine $D_{1}$ agonists produces sequential super-stereotypy of syntactic grooming chains (Berridge et al. 2005).

Electrophysiological rodent data showed that the response of dopamine neurons to the omission of an expected reward depends on a phasic decrease in the stimulation of $D_{1}$ receptors (Joel and Doljansky 2003). Such a decrease may disrupt switching to a different behavior, thus resulting in a repeated emission of the same behavior. Interestingly, the effect of a $D_{1}$ agonist on grooming in rodents was blocked by the $D_{2}$ receptor antagonist haloperidol (Joel and Doljansky 2003).

Another experimental rodent study showed that full stimulation of the dopamine $D_{1}$ receptor can increase the rate of transition through a stereotyped behavioral pattern. However, the mechanism through which dopamine facilitates this chain shortening is not clear (Matell et al. 2006). The timing of syntactic grooming phase transitions may involve a $D_{1}$-mediated internal clock process that is altered by full $D_{1}$ agonist activation. Thus, a full dopamine $D_{1}$ agonist might increase the speed of the clock used for the temporal control of grooming and shorten phase durations (Matell et al. 2006). The finding that dopamine receptor antagonists decrease the time spent engaged in repetitive behavior might help to explain why neuroleptics can be effective in treating OCD-like conditions (Hoffman 2012).

### Dopamine in repetitive thought and motor programs in humans

Binding potentials of $D_{1}$ receptors in caudate and putamen were found to be reduced in OCD patients compared with healthy controls (Olver et al. 2009). Reduced $D_{1}$ binding in the striatum of OCD patients may represent agonist-induced down-regulation of $D_{1}$ receptors secondary to an increased dopaminergic drive. These findings suggest that meso-cortical dopamine inputs via $D_{1}$ receptors may play a role in the etiology of OCD (Olver et al. 2009, 2010) although there is some methodological debate (Cervenka 2019).

Schizophrenia patients often have an impaired ability to shift response sets. This impaired shifting is accompanied by perseverative responses (Crider 1997). In some earlier studies, schizophrenic perseveration was viewed as the consequence of a task-inappropriate $D_{2}$ receptor-mediated re-selection of a previously activated cortico-striatal process (Crider 1997), or a consequence of low $D_{2}$ stimulation (Avery and Krichmar 2015).

Dopamine agonists, like amphetamine are well-known triggers of repetitive behavior, from simple motor movements to definitive compulsive behaviors (Zike et al. 2017, Denys et al. 2013). Atypical anti-psychotic medications, which act in part as dopamine receptor antagonists, may trigger obsessive-compulsive symptoms in some patients. Anti-dopamine D_1_ and D_2_ receptor antibodies are more frequent in patients with obsessive-compulsive symptoms (Cox et al. 2015; Endres et al. 2022).

A study with a selective radiolabeled ligand found that the total of $D_{1}$ and $D_{2}$ receptors in the caudate and putamen is not significantly different between schizophrenic patients and healthy controls (Sedvall et al. 1995). However, there was a significant reduction in the $D_{1}$ signal in high-intensity regions of the basal ganglia when a selective D_1_ antagonist ([11C]SCH 23390) was used (Sedvall et al. 1995). These results suggested reduced $D_{1}$-receptor density in the patch compartment of the basal ganglia in schizophrenic patients (Sedvall and Karlsson 1999). The low $D_{1}$ receptor density in this compartment may result in altered activity of the $D_{1}$/$D_{2}$ regulatory feedback system to limbic brain regions in schizophrenia (Sedvall and Karlsson 1999; Sedvall et al. 1995).

Related references of $D_{1}$ and $D_{2}$ receptor-mediated effects on stereotypy, action initiation and termination of repetitive behavioral sequences are presented in Table 1.

**Table 1:**
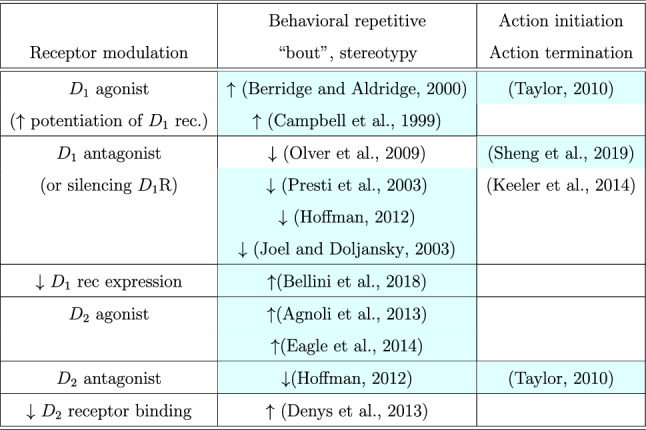
Related references of *D*_1_ and* D*_2_ receptor-mediated effects on the initiation and termination of repetitive motor and behavioral programs. Animal studies are highlighted, while human studies and reviews are not

**Fig. 1:**
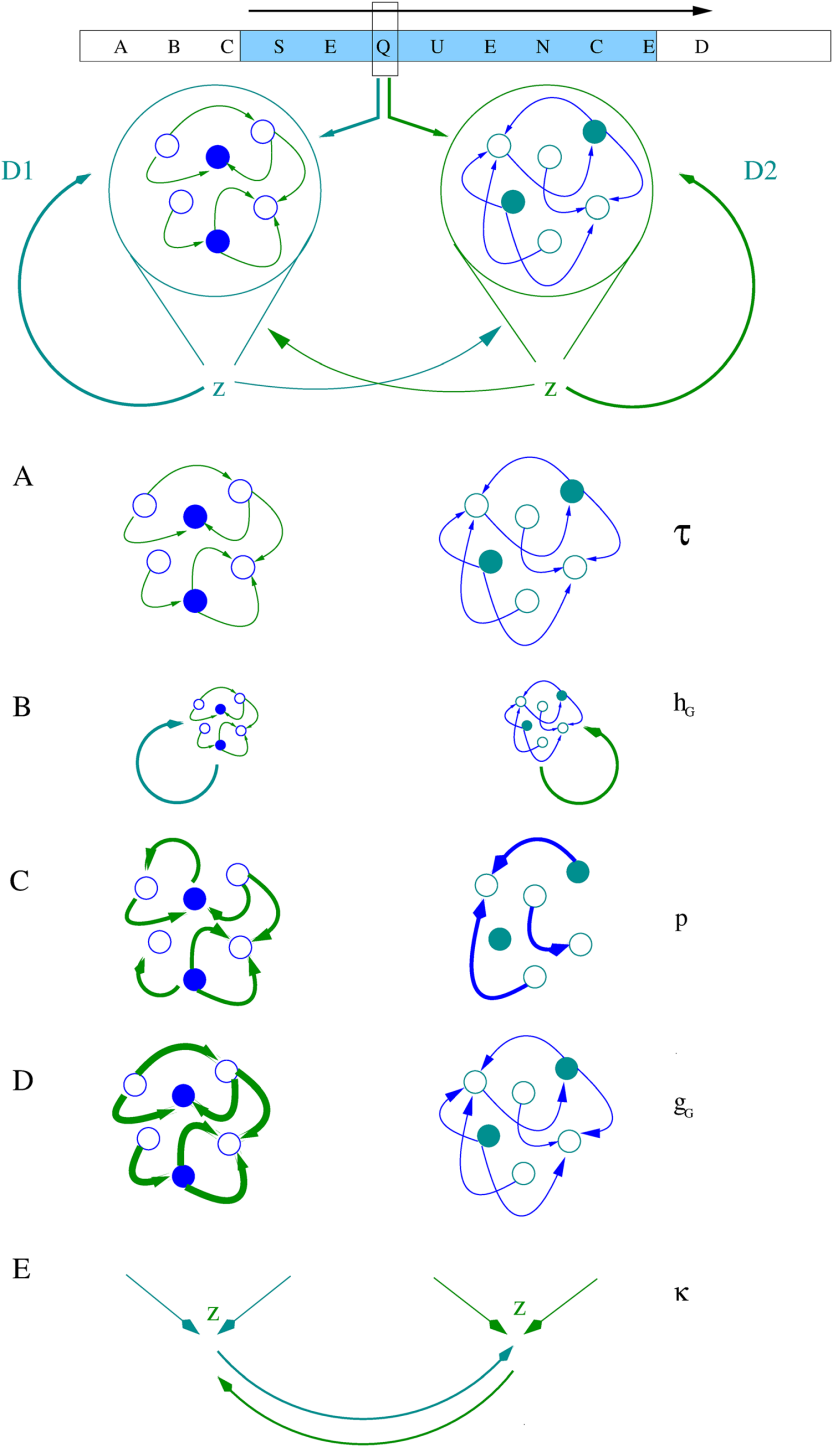
Schematic representation of the two-pool D_1_ and D_2_ medium spiny striatal network system. Parallel projections from the cortex provide sequenced input. Both striatal populations compute output which provides feedback teaching signals to itself and the other population (top panel). Z represents the output of readout units for each reservoir. **(A**) Experiment 1 looks at the effects of different time constants ($\tau$) in the two population sets. **(B**) Experiment 2 looks at the effects of self-feedback within each population ($h_{G}$). **(C)** Experiment 3 explores the effects of varying connection probabilities (p) of input connections, recurrent connections within reservoirs and the self-feedback matrix. **(D**) Experiment 4 investigates the effects of varying internal coupling strengths ($g_{G}$) in the reservoir networks. (**E**) Experiment 5 shows the effects of varying the coefficient ($\kappa$) of the teaching signal from the other reservoir

**Fig. 2:**
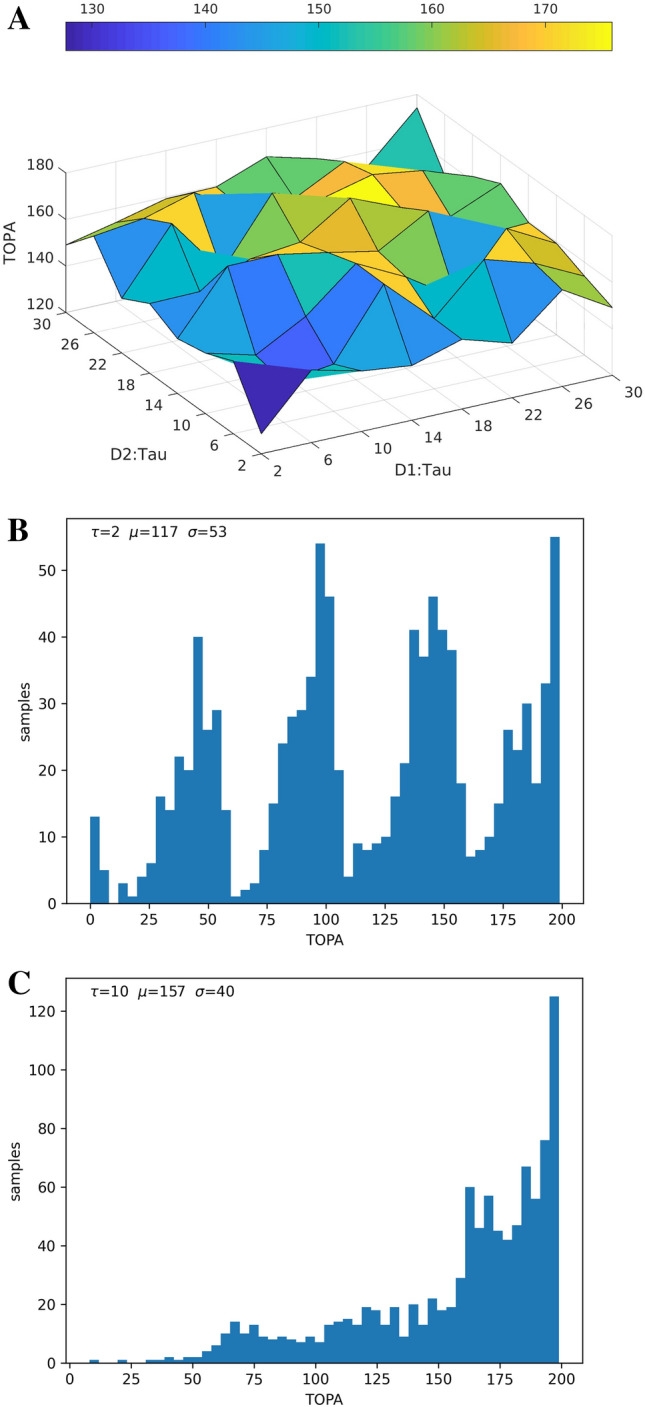
Varying the time constants ($\tau$ in ms) of the neural units in the two pools (Fig. 1.A). **(A)** Shows TOPA of readout units during chunk presentation for values of $\tau$ in both populations. **(B)** Shows a histogram distribution of TOPA with $\tau =2$  in both populations. **(C**) Shows a histogram distribution of TOPA with $\tau =10$ in both populations

**Fig. 3:**
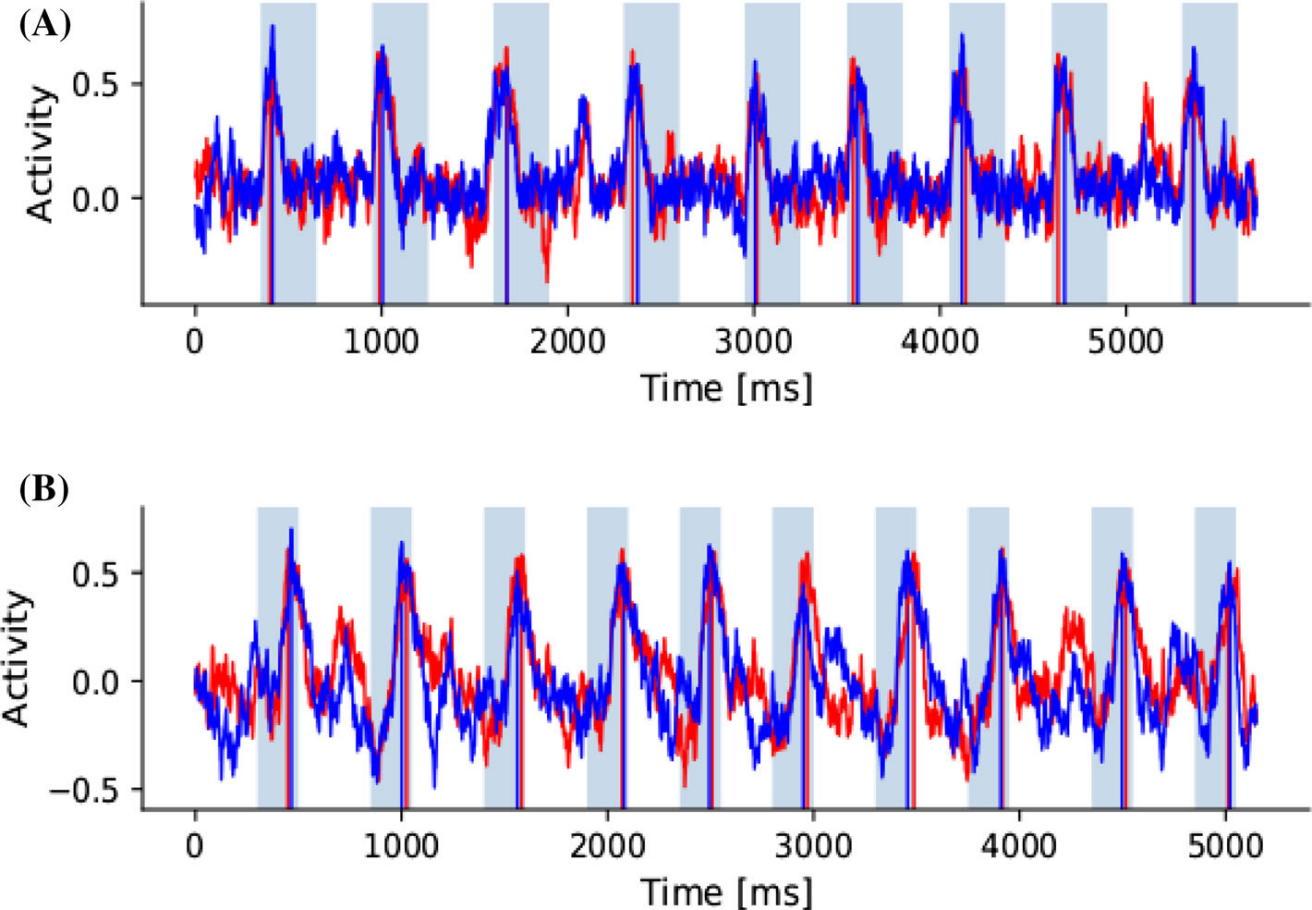
Examples of post-learning readouts of both populations. Shaded intervals indicate the chunk presentation periods, which the network was trained to recognize. The red and blue plots represent activity in each population. Vertical red and blue lines represent TOPA for each population within chunk periods. **(A)** Simulation when the time constants $\tau$ were selected as 2 and 6 ms in respective populations. **(B)** Time constants $\tau$ were 10 ms in both populations

## Computational Experiments

To improve understanding of how $D_{1}$ and $D_{2}$ asymmetries may contribute to the dynamics of sequence processing and disorders, eight computational experiments were performed with modifications to the reservoir computing model described by Asabuki et al. (2018). The first experiment investigated the effects of varying MSN response time constants. The second set of simulations explored the self-feedback to each MSN population. The third experiment studied the effects of varying connection probabilities. The fourth simulation set examined the effect of varying weights within the $D_{1}$ and $D_{2}$ receptor mediated MSN populations. In the fifth experiment, we investigated the effects of assymetric feedback between the $D_{1}$ and $D_{2}$ receptor dominated MSN populations. The sixth simulation set examined the effects of an increased input chunk size. In the seventh experiment, a combination of 3 separate parameters were altered. Those parameters were chosen from values which contributed separately to earlier chunk recognition. Finally, in the eighth simulation set we studied the effects of variations in learning rate and training times on chunk recognition. Throughout the computational experiments, time offset of the peak activity (TOPA) was calculated, which can represent the recognition of the chunk. The first five experiments are illustrated in Fig. 1.

### 1: Varying the time constants of the reservoir units

There is an anatomical and physiological dichotomy between $D_{1}$ and $D_{2}$ receptor expressing MSNs. Electrophysiological measurements have demonstrated differential single cell excitability and membrane time constants, as well as differential channel composition and variations in morphology (Gertler et al. 2008). $D_{2}$ MSNs have a shorter membrane time constant than $D_{1}$ MSNs and it is accepted that the $D_{1}$ MSNs are less excitable than $D_{2}$ MSNs. Identical synaptic events generate smaller excitatory post-synaptic potentials (EPSPs) in $D_{1}$ MSNs than $D_{2}$ MSNs. Injecting current into dendrites of $D_{1}$ MSNs was also less effective in generating repetitive spiking than in the $D_{2}$ MSNs (Gertler et al. 2008). Given these motivations, we investigated the effects of varying time constants in the striatal $D_{1}$ and $D_{2}$ MSN populations (Fig. 1.A.).

### 2: Self-feedback within $D_{1}$ and $D_{2}$ MSN populations

This simulation set explored the effects of collective self-feedback within each MSN population (Burke et al. 2017) (Fig. 1.B.). Cholinergic interneurons modulate the excitability of spiny neurons. They span large regions of the striatum and receive inputs from many MSNs. This feedback control system allows cholinergic neuronal dynamics to influence both the $D_{1}$ and $D_{2}$ MSNs.

### 3: Connection probabilities of the MSN reservoirs

The intra-striatal connectivity between striatal projection cells modulates neuronal firing and shapes the output of the circuit (Wickens et al. 2007; Burke et al. 2017). A striatal study used a connectivity value of 20% based on mean data from several different laboratories (Wickens et al. 2007). Intrastriatal MSN $\rightarrow$ MSN connections have shown asymmetries. Experimental rodent work showed a 7% connection probability between D_1_ MSNs and a 23% connection probability between D_2_ MSNs (Planert et al. 2010). Computational studies based on experimental results used a weaker D_1_ $MSN$ $\rightarrow$D_1_ $MSN$ than D_2_ $MSN$ $\rightarrow$ D_2_ $MSN$ coupling (Bahuguna et al. 2015, 2019). We examined the effects of simultaneously varying connection probabilities within the two parallel reservoirs (p) (Fig. 1.C.), input projections to reservoir units and non-zero weights in the self-feedback weight matrices, represented collectively as p3.

### 4: Weights of the internal connections within the MSN reservoirs

In the next set of simulations, internal coupling strengths were altered in the reservoirs ($g_{G}$), while keeping the connection probabilities constant. The strength of the intrastriatal synaptic connections are influenced by dopaminergic input and pathologies. We investigated the effect of varying the internal coupling strength on TOPA during chunk presentation (Fig. 1.D.).

### 5: Feedback between $D_{1}$ and $D_{2}$ MSN populations

There is an experimentally observed asymmetry of recurrent and mutual connections among the two types of MSNs in the striatum (Planert et al. 2013; Taverna et al. 2008). In the original modeling study, the teaching signals were chosen to be symmetric with respect to the interchange of the two readout units. This was determined such that the two systems stop training when both readout units output similar response patterns (Asabuki et al. 2018). However, the connection probability from $D_{2}$ $\rightarrow$ $D_{1}$ MSNs is higher (Planert et al. 2013; Taverna et al. 2008). Thus, the $D_{1}$ MSNs receive a higher number of stronger synapses from $D_{2}$ MSNs than vice versa (Planert et al. 2010). High amplitude GABA currents have been recorded for depolarized MSNs, which is coherent with a strong hyperpolarizing effect, but a depolarizing effect of GABA has also been previously described in striatum (Mercuri et al. 1991), (Szalisznyó and Érdi 2000). GABA might have a shunting inhibition effect also (Fino et al. 2018).

Accordingly, the effects of varying the teaching signal coefficient ($\kappa$) between the two populations was explored. The activation function (implemented as $tanh$ in the model) is allowed to become both negative and positive. Negative activation may correlate with shunting inhibition between inhibitory populations as well. The teaching signal from contra populations can then become inhibitory and result in competitive dynamics (Fig. 1.E.).

### 6: Increasing the chunk size

To investigate the sensitivity of start and stop signaling on chunk size, the length of the chunks were increased from four to six characters. Other simulation parameters were unchanged except for time constants, which were varied in both reservoirs as in experiment 1.

### 7: Combining parameters from experiments 1, 2 and 4

Given the previously described separate parameter sensitivities in experiments 1, 2 and 4, parameters were selected for early chunk recognition and combined for this simulation. The $g_{G}$ and $h_{G}$ values were chosen to be 0.5 and 0.4 respectively. The time constant parameter space was explored, as in experiments 1 and 6.

### 8: Effects of learning rate and training time on chunk learning

The model learning rate (α) represents neural plasticity and training time represents previous exposure to chunks during learning. The TOPA z-score is a representation of peak signal strength and an indication of how well the reservoirs learned to recognize the chunks during training. The training time and learning rate were independently varied to investigate effects on TOPA z-score.

**Fig. 4:**
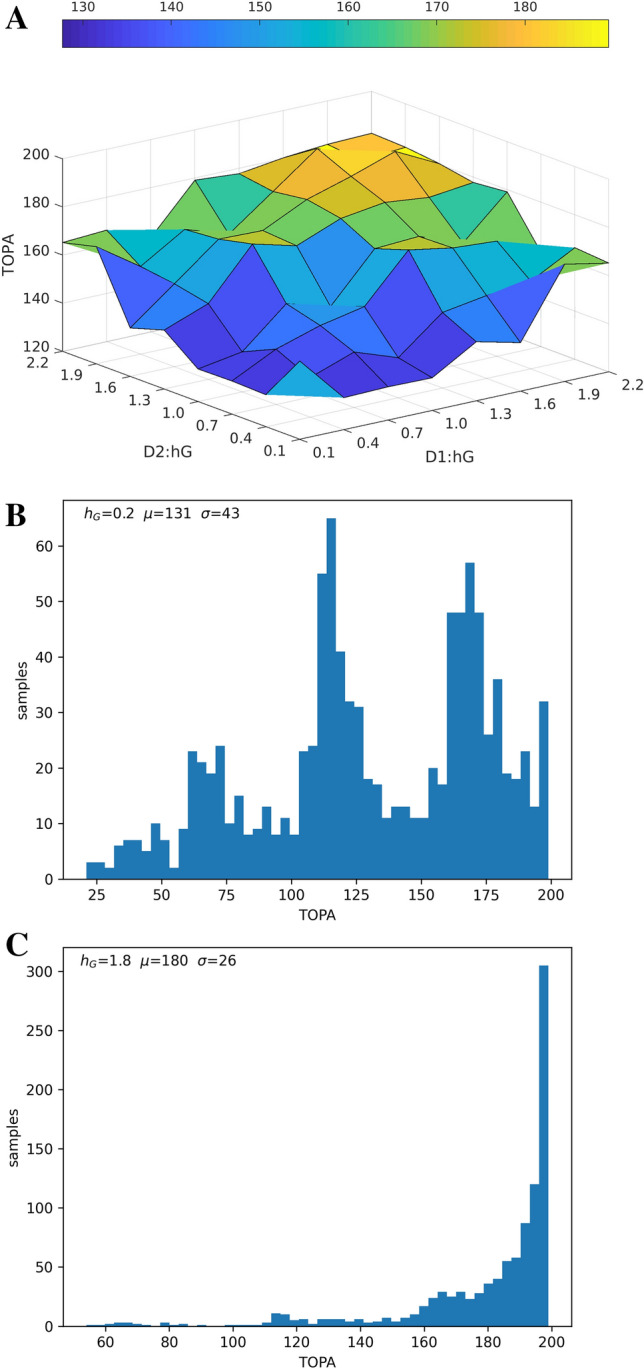
Varying the recurrent self-feedback gain $h_{G}$ (Fig. 1.B.) in both populations. **(A)** Shows that with higher feedback TOPA is shifted towards later parts of the chunk. (**B**) Shows a histogram distribution of peak activity with $h_{G}=0.2$ for both populations. (**C**) Shows a distribution with $h_{G}=1.8$ for both populations

**Fig. 5:**
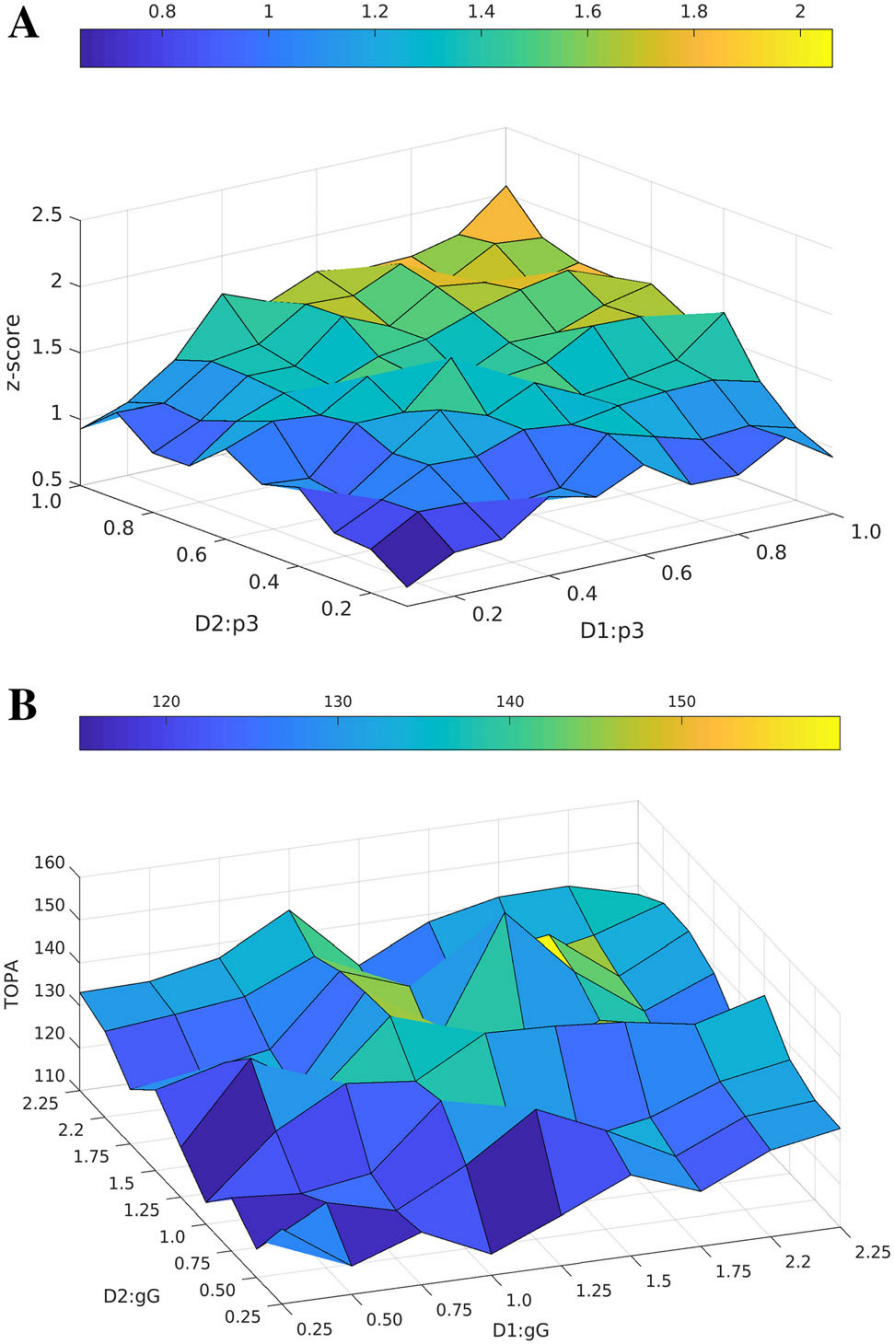
(**A)** Shows simultaneously varying connection probabilities (p3) of input projections, internal unit connections and self-feedback matrices on both reservoirs (Fig. 1.C.). The z-score on the Z axis represents the statistical significance of TOPA occurrence. (**B**) Shows TOPA non-monotonicity when varying the strengths of connections $g_{G}$ of internal units

**Fig. 6:**
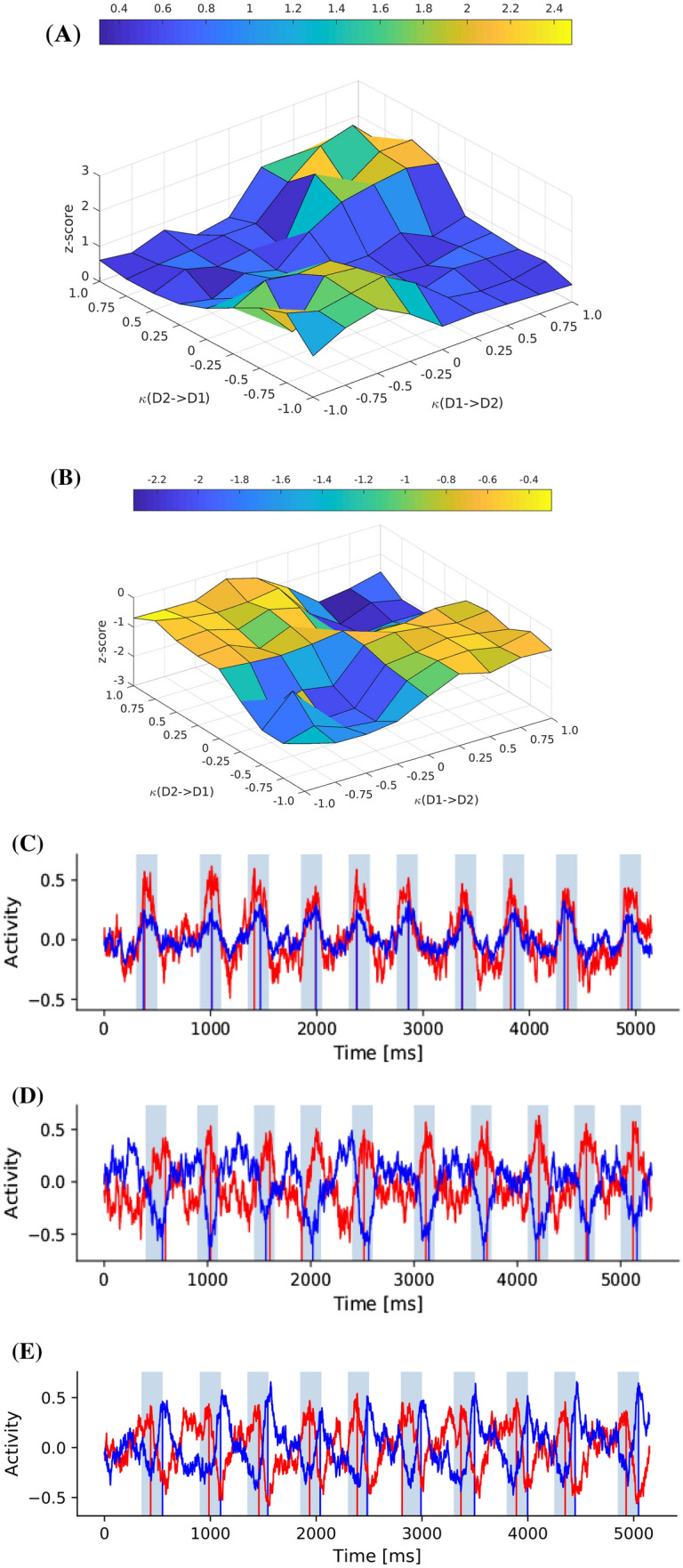
Varying kappa $$\kappa$$, the teaching signal coefficient from the contra reservoir. The z-score represents the statistical significance of TOPA occurrence. A z-score approaching zero is near baseline (mean) readout activity during a simulation trial. (**A**) TOPA significance in the population which emerges as excitatory. (**B**) TOPA significance in population which emerges as inhibitory. (**C**) Shows readout dynamics with $$\kappa = 1
$$ for one reservoir in blue and 0.5 for the other in red, showing a lower activation amplitude. (**D**) An emergent example with $$\kappa = -1
$$ for both reservoirs, producing competitive effects. (**E**) Another example with $$\kappa = -1$$ in both reservoirs, showing possible activity similar to start/stop cells

## Model description

We adapted the modeling framework published and specified previously by (Asabuki et al. 2018). The following equation was employed for the two reservoirs:

1 $$\begin{aligned} \tau \frac{dx_{i}}{dt}=  -x_{i}(t) + g_{G} \sum ^{N_{G}}_{j=1} J^{GG}_{ij}r_{j}(t) & + h_{G}J_{i}^{GZ}z(t) \\& + \sum ^{N_{I}}_{\mu =1}J_{i\mu}^{GI} I_{\mu }(t) + \sigma \xi _{i}(t) \end{aligned}.$$

where each reservoir is composed of N_*G*_ neurons with *i=1,2,...N*_*G*_. The dynamics of each reservoir neuron is represented by *x*. $I_{\mu }(t)$ represents the activity of the inputs and N_I_ is the number of input neurons. $\xi _{i}(t)$ denotes the random (Wiener) process and $\sigma$ is the standard deviation. $J^{GG}$, $J^{GZ}$ and $J^{GI}$ describe the recurrent internal connection matrix of the reservoir, the self-feedback matrix and the matrix between input and the reservoir neurons, respectively. The recurrent internal and self-feedback connections are non-plastic. The parameter $g_{G}$ scales the recurrent internal weight matrices. The parameter h_G _ scales the self-feedback weight matrices. The activation function is defined as: 

2 $$\begin{aligned} r_{i}(t)=tanh(x_{i}(t)). \end{aligned}$$

The instantaneous output is given by *z*(*t*), where *w* is the readout weight vector:

3 $$\begin{aligned} z(t)=w^Tr(t). \end{aligned}$$

The readout unit is connected with *n* reservoir neurons by the weight vector *w,* which is trained using the FORCE learning algorithm (Sussillo et al. 2009). Weights *w* undergo learning with a teaching signal given by the output of the partner network. The teaching signal is described according to:

4 $$\begin{aligned} f_{i}(t)=[tanh({\widehat{z}}_{j}(t)/\beta )]_{+}. \quad (i,j=1,2; \quad i\neq j) \end{aligned}$$

where ${\widehat{z}}_{j}(t)$ is the normalized output of a readout unit (see in methods in Asabuki et al.), the threshold linear function $[x]_{+}$ returns 0 if *x* $\leqq$ 0, and $[x]_{+} = x$ if $x>0$. Note that in simulation set 5 this constraint was removed, such that function* f *can go negative. The constant $\beta$ = 3 was set as in the original work (Asabuki et al. 2018). The error signal was defined as:

5 $$\begin{aligned} e_{i}(t)=z_{i}(t)-\kappa f_{i}(t). \quad (i=1,2)    \end{aligned}$$

The parameter $\kappa$ was 1 in all experiments except in simulation set 5, where it was varied between $-1$ and 1. For initial values of weight matrices, see methods in (Asabuki et al. 2018). The connection probability* p *of reservoir neurons is 1 in all experiments except 3, 4 and 5. In experiments 3 and 4, the parameter *p3* indicates a common connection probability in matrices J^GG^, J^GZ^ and J^GI^. Parameter values are summarized in Table 2 for each experiment.

### Definition of time offset of peak activity (TOPA)

The maximum post-training activity values (peak) of readout units during the chunk presentation periods were calculated for every simulation. The time position of this peak activity after the start of the presented chunk was determined for every simulation and is denoted as TOPA. For example, Fig. 3 shows activity plots for each readout unit with vertical lines marking TOPA. For experiment 5, the minimum peak was also calculated.

**Fig. 7:**
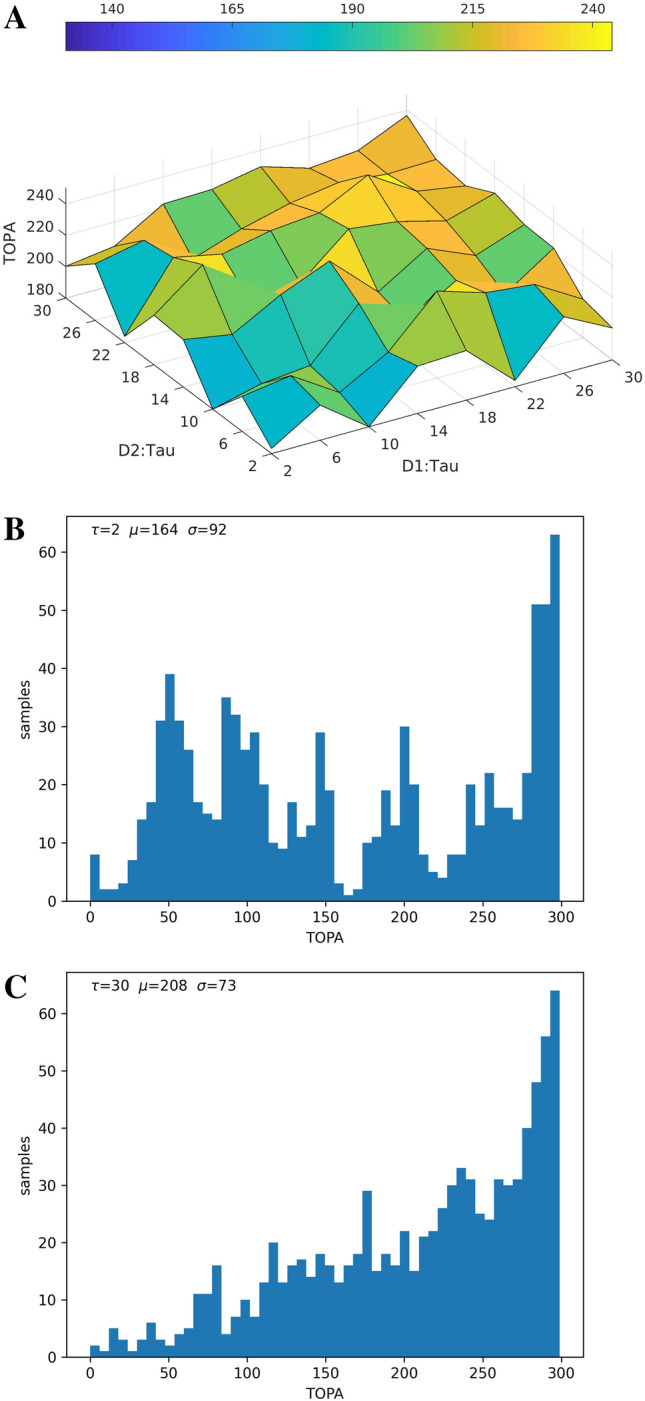
Varying the time constants (τ in ms) of neural units in the two pools with an increased chunk length of 6 characters (from 4). (**A)** Shows TOPA while varying $\tau$ in both reservoirs during chunk presentation. (**B)** Histogram distribution of TOPA with $\tau=2$ in both populations. (**C)** Histogram of TOPA with $\tau = 30$ in both populations

**Fig. 8:**
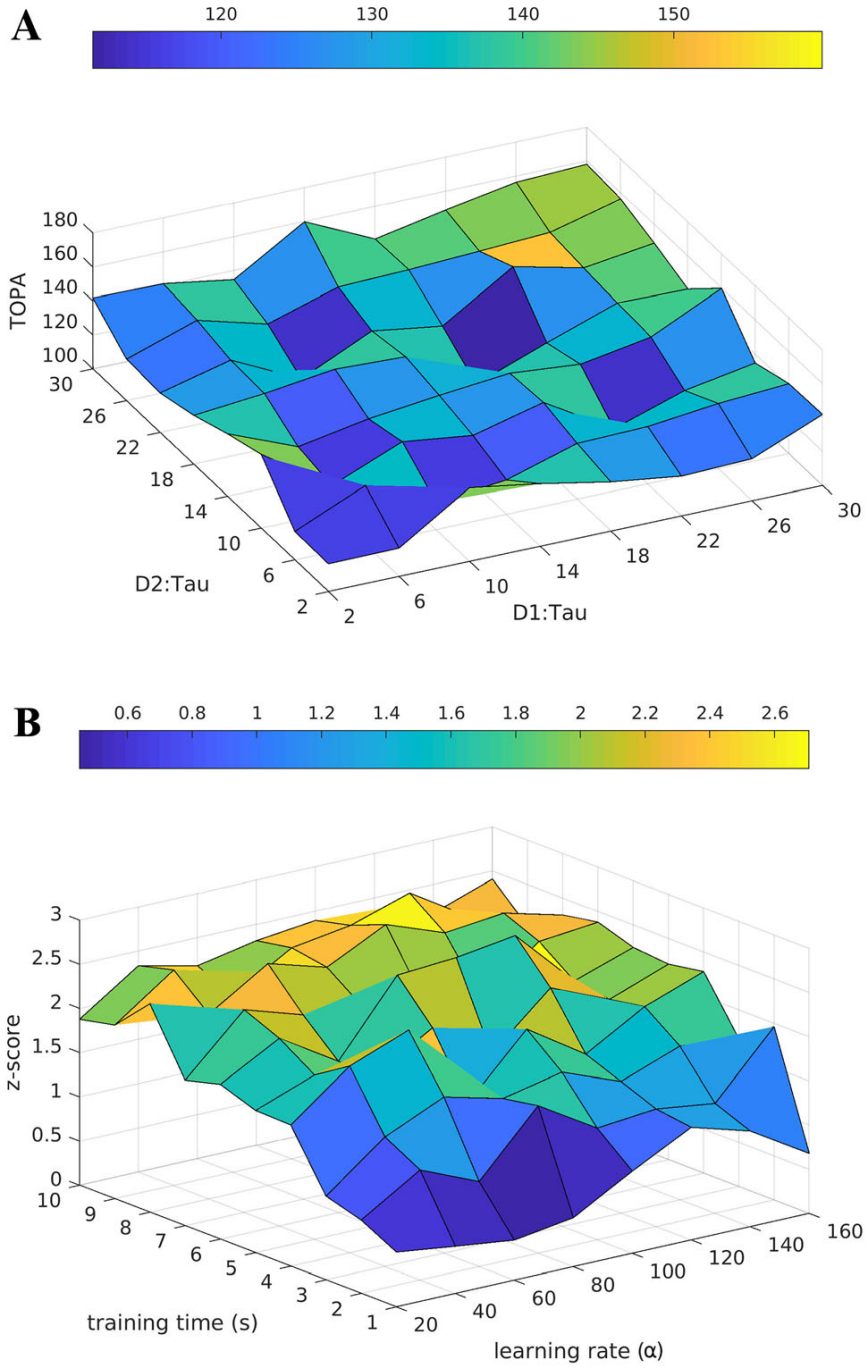
(**A**) Varying the time constants (τ in ms) of neural units in the two pools while combining parameter values from experiment 2 and 4, selected for contribution to early chunk recognition $h_{G}=0.4$, $g_{G}=0.5$. (**B**) Effects of varying the learning rate and training time on the TOPA z-score during chunk recognition

**Fig. 9:**
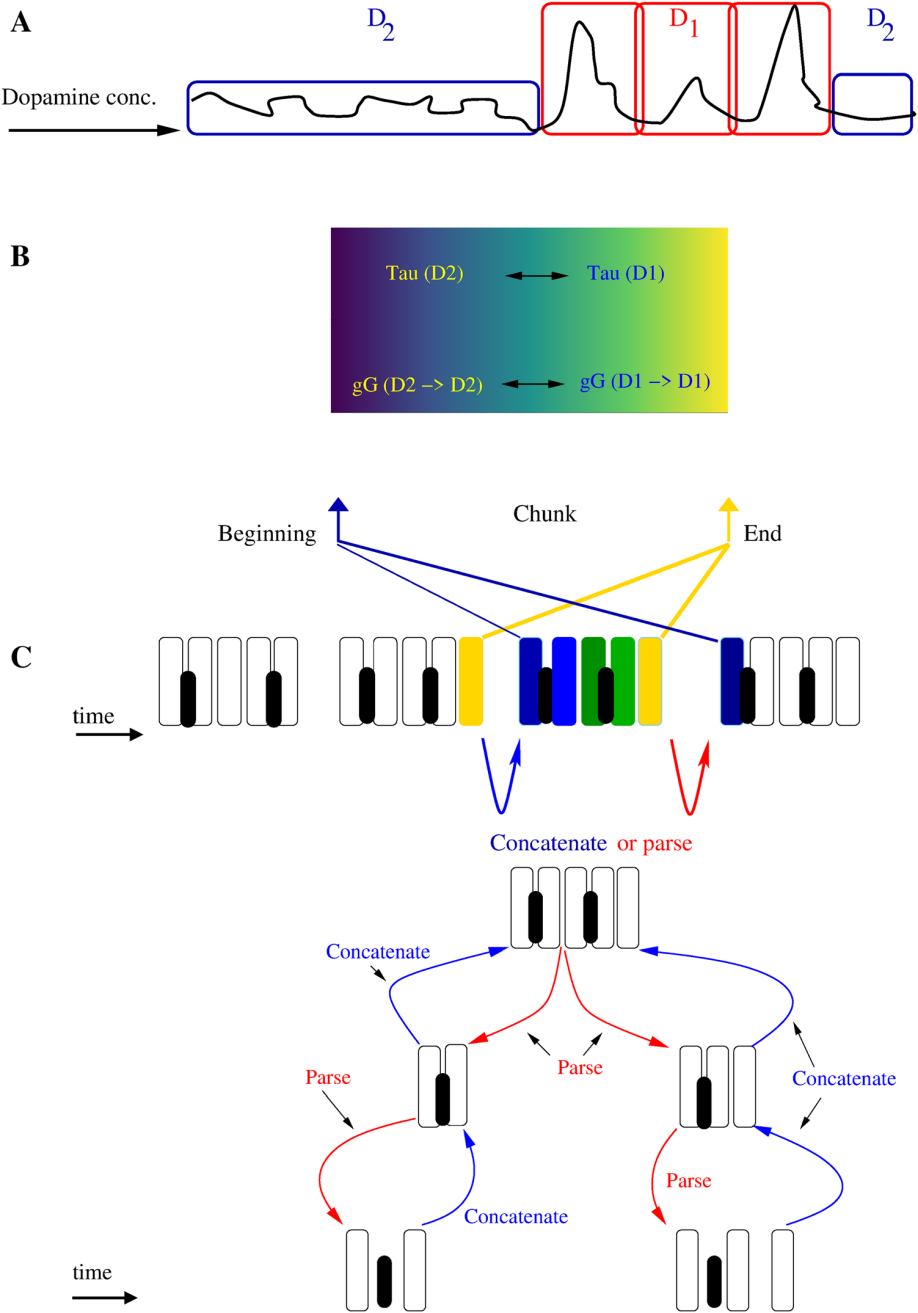
Schematic representation of the hierarchical parsing and concatenation of sequences. (**A**) Shows $D_{1}$ and $D_{2}$ receptor responses to varying dopamine concentrations over activity and time. It illustrates that $D_{1}$ receptors are preferentially stimulated by phasically released dopamine, whereas low-level baseline tonic dopamine release is sufficient for stimulating $D_{2}$ receptors (Schultz 2007). (**B**) Summarizes some of the investigated parameter contributions towards recognizing beginnings and endings of the chunks. (**C**) Illustrates the signaling of the chunk boundaries. Sequences can be concatenated into an single integrated sequence or a single chunk can be decomposed into constituents. We suggest that $D_{1}$ and $D_{2}$ receptor mediated dynamical boundary signaling may influence hierarchical chunk learning and processing

## Numerics

Simulations were performed on a compute cluster at the UPPMAX supercomputer center at Uppsala University. Simulations used python 3.8 and had a timestep of 1 ms. Simulations were averaged over 10 different seeds for each parameter regime.

**Table 2:** Summary of the parameters used in simulation experiments

Parameter	Exp.1 Fig.2.	Exp.2 Fig.4.	Exp.3 Fig.5.A.	Exp.4 Fig.5.B.	Exp.5 Fig.6.	Exp.6 Fig.7.	Exp.7 Fig.8.A.	Exp.8 Fig.8.B.
$N_{G}$	300	300	300	600	300	300	300	300
$n$	300	300	300	300	300	300	300	300
$\sigma$	0.3	0.3	0.3	0.3	0.3	0.3	0.3	0.3
$\beta$	3	3	3	3	3	3	3	3
$\tau_{1},\tau_{2}$	2-30	2-30	10,10	10,10	10,10	2-30	2-30	10,10
$h_{G}$	1	0.1-2.2	1	1	1	1	0.4	1
$p$	1	1	0.1-1.0	0.3	0.25	1	1	1
$p3$	1	1	0.1-1.0	0.3	1	1	1	1
$g_{G}$	1.5	1.5	1.5	0.25-2.25	1.5	1.5	0.5	1.5
$k$	1	1	1	1	–1 to 1	1	1	1
$chunksize$	4	4	4	4	4	6	4	4
$\alpha$	100	100	100	100	100	100	100	20-160

## Results

In simulation experiment 1, we examined the effects of varying time constants ($\tau$) in the two parallel MSN networks (Fig. 1.A., Fig. 2., Fig.3.). The time constants of each population were independently varied between 2 and 30 ms.

We found that neural activity often peaked earlier during the chunk when the time constants of the reservoir were shorter, perhaps similar to the response of Start cells (Figs. 2.A., B, 3.A.). With increased time constant values, the peak activity shifted more often towards the end of the chunk, perhaps similar to Stop cells (Fig. 2.C., 3.B.). This modeling result implies that asymmetric time constants of the two parallel $D_{1}$ and $D_{2}$ dominated MSNs may contribute to differential signaling at the start and stop positions in chunks. The distributions of peak activity (TOPA) are represented as histograms in Fig 2. With short time constants, TOPA may occur during leading chunk segments, as can be seen with four clusters in the histogram, likely corresponding to presented chunk characters (Fig. 2.B). With longer time constants, TOPA occurs most often at the end of chunk presentation (Fig. 2.C).

In computational experiment 2, we altered the self-feedback gain ($h_{G}$) within the reservoir networks (Fig. 1.B.). A monotonic increase was observed as the gain $h_{G}$ increased. With lower self-feedback gain (or weaker feedback coupling), the peak activity more often signals the beginning of the chunk. As self-feedback gain increased, peak activity shifted towards the end of the chunk (Fig. 4.A.). Histograms show that with low feedback gain, TOPAs can occur earlier in chunks (Fig. 4.B.), while with stronger feedback, TOPAs accumulate at the end of chunks (Fig. 4.C.). This implies that TOPAs are sensitive to the contributions of previous states, depending on time constants. Note that in Fig. 4.A. TOPA was averaged across both populations, but treated as separate samples in Fig. 4.B and C.

In simulation experiment 3, the effects of varying combined connection probabilities (*p3*) were examined (Fig. 5.A.). The connection probability was varied between 0.1 and 1 on input projections to reservoir units, internal recurrent connections within the reservoirs themselves and the weights in the self-feedback matrix. When *p3* is low, TOPAs during chunk presentation occured with lower z-scores, indicating a weaker signal strength. As *p3* increases, TOPA z-scores gradually increase, indicating higher statistical significance with denser connectivity (Fig. 5.A.). The z-score represents the number of standard deviations away from mean activity of the reservoirs.

In simulation experiment 4, the effects of scaling the intrinsic weight matrix was analyzed ($g_{G}$) (Fig. 1.D.). This simulation used *p3* = 0.3 for connection probabilities. A non-monotonicity was observed as the gain of the intrinsic weight matrix was varied (Fig. 5.B.), with higher values the network is more likely to signal the end of the chunk. The highest TOPA was observed at g_G_ = 1.5 for both populations, which also had the highest z-score (not shown). These results suggest that weaker D_1_ $MSN$ $\rightarrow$ D_1_ $MSN$ (Planert et al. 2010; Taverna et al. 2008) may result in network activity which more often signals the beginning of chunk while stronger D_2_ $MSN$ $\rightarrow$ D_2_ $MSN$ connections more often contribute to signaling the end of chunks (Fig. 5.B.).

In simulation experiment 5, the reservoir teaching signals were investigated, which originate from the opposing population. During training, the differences in activity levels are used as error signals to adjust the readout weights. This experiment included coefficients $\kappa$ for teaching signals from the opposite reservoir and independently varied them from -1 to 1. On activation peaks during chunk recognition, a z-score was computed. In the baseline case $\kappa$ is 1 on both teaching signals, which result in the reservoirs seeking to achieve a consensus. When $\kappa$ is -1 on both teaching signals, the reservoirs compete, perhaps as go and no-go pathways do. Fig. 6.A. shows the positive z-scores when activation peaks are positive and Fig. 6.B. shows the negative z-scores when activation peaks are negative. Low z-scores approach the mean population activity levels. When the teaching signals have opposing signs, the reservoirs do not learn properly, and the z-scores are near the mean. The corners where the signs are the same show either collaborative ($\kappa$ = 1) or competitive ($\kappa$ = -1) activations. Fig. 6.C. is a single trial that shows one population in red with $\kappa$ = 1 and the other in blue with $\kappa$ = 0.5. Fig. 6.D. is one emergent variation with $\kappa$ = -1 and competitive dynamics. Fig. 6.E. is another emergent variation with $\kappa$ = -1 that shows one population in red peaking at the beginning of the chunks and the other population in blue peaking at the ending of the chunks. These dynamics may indicate that some level of competition is necessary between populations to achieve start/stop chunk signaling.

In simulation experiment 6, the size of the chunk was increased from 4 to 6 characters. As in experiment 1, the $$\tau _{1}$$ and $$\tau _{2}$$ parameter space was explored. Small $$\tau
$$ values again support early peak activity on partial chunk recognition (Fig. 7.A.), which disappear with longer time constants (Fig. 7.B., C.). The distribution histogam in Fig. 7.B. at $$\tau = 2$$ in both populations now shows 6 clusters rather than the original 4, likely corresponding with the additional presented chunk characters. The histogram in Fig. 7.C. has $$\tau =30$$ in both populations, showing a distribution gradient peaking at the presentation of the last chunk character. These results indicate that time constants (τ) may contribute to start/stop signaling at least partially independent of chunk size.

In simulation experiment 7, the combined parameter space results in recognition of the earlier part of the chunk while the basic structure of the previously observed tendency is preserved, such that shorter time constants result in earlier chunk recognition (Fig. 8.A.). The nonmonotonicity in the 2-dimensional surface reflects the chunk segments, having 4 characters.

Simulation experiment 8 examined the learning properties of the model with $\tau =10$ in both populations, by varying the training times from 1 to 10 s (baseline) and the learning rate $\alpha$ from 20 to 160 (100 in baseline). Fig. 8.B. shows that training converged to a maximum learning capability at a learning rate of α = 100 and training time of 10 s.

## Discussion

We propose that D_1_ and D_2_ receptor-dominated striatal MSN dynamics can contribute to start and stop signaling cues and thus enable a chunking strategy which improves performance in sequence processing (Solopchuk et al. 2016). In OCD and schizophrenia, this chunking strategy is altered, which can give rise to some of the overlapping symptomatology. Chunking during sequence learning is a dopamine-dependent process (Tremblay et al. 2010; Taylor 2010). In a rodent study, the D_1_ and D_2_ MSNs showed different patterns of lick sequence-related activity and different phases of oscillation time-locked to the lick cycle, both at coarse and fine timescales (Chen et al. 2021). A D_2_ receptor antagonist has been shown to have deleterious effects on the chunking of separate movements into integrated motor sequences in monkeys (Tremblay et al. 2009). Perseverative errors in schizophrenia result in continued re-selection of previously activated outputs (Yogev et al. 2003; Szalisznyó et al. 2019). Over-switching is a counterpart of perseveration, and both can be characteristic of schizophrenia and OCD. The underlying D_1_ and D_2_ receptor pathology could contribute to parsing and concatenation error in these conditions (Fig. 9.) (Yogev et al. 2003). Compared with normal performance of the same motor tasks, OCD rituals are longer in duration and comprise a greater repertoire of idiosyncratic (unnecessary) acts (Eilam 2017). A human study showed that baseline striatal D_2_/D_3_ receptor binding is decreased in OCD, supporting the hypothesis of chronically increased endogenous dopaminergic activity (Denys et al. 2013).

D_1_ receptors have been demonstrated to have a greater sensitivity for phasic dopamine transmission (Dreyer et al. 2010). On the other hand, D_2_ activation might facilitate switching between conflicting mental representations (Bensmann et al. 2020; Agnoli et al. 2013). The literature is controversial on whether the D_1_ and D_2_ MSNs signal more initiation or termination of sequences. D_1_ neurons are more relevant to cue perception and initiation of specific motor action, whereas D_2_ neurons are more involved in post-movement events (Sheng et al. 2019). One rodent study (Jin et al. 2014) implicated that the initiation vs. execution of actions involve different subsets of D_1_ and D_2_ MSNs. When the MSN neurons were subdivided, a similar percentage of D_1_ MSNs signaled the sequence start vs. stop, while the majority of D_2_ MSNs preferentially displayed activity related to the start rather than the end of the sequence (Jin et al. 2014). Another rodent study demonstrated that the major role of D_2_ MSNs is in action initiation (Augustin et al. 2020). In the following sections, related literature is discussed in the context of our modeling results.

### Neuropathological correlates related to the simulation results

#### Time constants and excitability

Dopamine has opposite effects on excitability of D_1_ and D_2_ MSNs (Planert et al. 2013). Phosphodiesterase 10A (PDE10A) is a unique postsynaptic signaling molecule located mainly in the striatal MSNs, regulating neuronal excitability. Alteration of this striatal enzyme was associated with schizophrenia symptoms (Bodén et al. 2017). The decreased striatal PDE10A concentration in schizophrenia patients may correlate with increased MSN excitability. As MSNs become more depolarized, even small inputs can cause neurons to fire. The signal-to-noise ratio may drop, making the system more susceptible to noise (Bodén et al. 2017; Persson et al. 2020). PDE10A mediates salience if reward-predicting cues are impaired in PDE10A knockout mice performing reinforcement tasks (Piccart et al. 2014). In schizophrenia, elevation in D_2_ receptor density and occupancy is observed in the striatum of drug-free and drug-naive patients (Laruelle 1998; Simpson 2010). Recognizing chunks prematurely may contribute to positive symptoms.

A rodent study demonstrated that chronic upregulation of D_2_ receptors increases the excitability of MSNs via downregulated expression of inward rectifier potassium channels (Kir). Changes in excitability of MSNs may impair filtering (Cazorla et al. 2012). These findings are in agreement with our results in experiment set 1, where the D_2_ mediated dynamics could more likely signal start phases, due to shorter membrane time constants, thus faster single neuron dynamics. However, in vivo studies suggest that D_2_ MSNs convey a powerful stop signal. This signaling is most likely implemented via the sub-thalamic nucleus to the substantia nigra pars reticulata, thus via larger network effects (Garr 2019; Klaus et al. 2019), which this current model cannot capture. Electrophysiological properties differ between MSN subtypes, with D_2_ MSNs exhibiting increased intrinsic excitability compared with D_1_ MSNs (Gertler et al. 2008). However, it should be noted that electrophysiological properties (e.g. membrane time constants), vary in the literature, depending on animal species, striatal regions, the electrophysiological metric and perhaps age (Willett et al. 2019; Planert et al. 2010; Planert et al. 2013).

#### Altered self-feedback gain

We explored the effects of varying the self-feedback within each population (Franklin and Frank 2015; Burke et al. 2017). The D_1_ and D_2_ receptors differently modulate the cholinergic interneuron excitability (Szalisznyó and Müller 2009). Since cholinergic neurons receive inputs from the MSNs and provide feedback by acutely modulating MSN excitability and synaptic function, as well as controlling striatal plasticity, we regard the feedback parameter to represent the activity of these cholinergic interneurons, which were not modeled explicitly. Experiment set 2 showed that with increased feedback the network is more likely to signal towards the end of the chunk (Fig. 4.A.).

#### Intrastriatal connection strengths

MSN collateral axon terminals are under dopaminergic regulation which can become dysfunctional when the dopaminergic innervation changes. A rodent study demonstrated that D_1_ and D_2_ MSNs form high-rate, one-way collateral connections with a homotypic preference. Physiologically, the D_2_ MSN → D_2_ MSN coupling is stronger than the D_1_ MSN → D_1_ MSN connections. Chronic D_2_ receptor activation results in a greater synaptic efficacy via a coordinated increase of synaptic GABA_A_ receptor clusters and GABA release sites (Lalchandani et al. 2013). However, another study demonstrated that overexpression of dopamine D_2_ receptors in MSNs decrease the complexity and length of their dendritic arbors (Cazorla et al. 2012). Decreased arborization is further associated with increased electrical excitability, due to a reduction of inward rectifier potassium currents (Cazorla et al. 2012). The methodological differences between the above studies might contribute to the divergent results on dendritic arborization changes. Simulation results in experiment 4 are more consistent with this latter finding that decreased arborization occurs from overexpression of D_2_ receptors (Cazorla et al. 2012), which may be associated with decreased connection strength. This, together with increased neural excitability, can shift the D_2_ MSN population towards recognizing the beginning of the chunks.

#### Other computational studies

A recent computational study found that when two parallel reservoirs were operating while using identical parameters, the performance of the system cannot be further improved. By setting suitable mismatched parameters between the two reservoirs, better prediction performance and higher memory capacity could be achieved. The two parallel reservoirs could increase the data processing rate as well (Yue et al. 2021).

It has been suggested that setting leaking rates differently for different network units (e.g., by splitting them to several sub-populations with constant value) can help in multi-timescale tasks (Lukosevicius 2012; Szalisznyó et al. 2017). Another computational study predicted that both low D_1_ and/or high D_2_ states can result in perseverative errors (Avery and Krichmar 2015). It was shown in cortical circuits that heterogeneous encoding of the input allows flexible learning resulting from the variety of timescales present in the reservoir (Bernacchia et al. 2011). Our current study shows that having different parameters in the two parallel MSN reservoir networks contributes to an improved signaling of the beginning or end positions of the presented and learned chunks.

## Limitations of the study

We used an abstract and mechanistic model to represent the $D_{1}$ and $D_{2}$ MSN population dynamics. We did not take into account the certain overlap of these two neuronal pools. Further, the feedback from the striatum to the cortex via the cortico-striato-thalamo-cortical neural pathways is not represented in the current study. We did not take into account the fact that $D_{1}$ receptor activation of working memory in the prefrontal cortex occurs at lower concentrations than needed for the modulation of motor function, thus the phasic or tonic distinction is an oversimplification. The temporal dynamics of the dopaminergic input is also excluded which carries an important dynamical modulation and opens several other avenues for future work. A recent modeling study challenged the view that differences in receptor affinity introduce asymmetries in $D_{1}$ and $D_{2}$ signaling and proposed that both pathways respond to the whole range of dopamine signals and integrate the dopamine signal over longer time scales (Hunger et al. 2020). Further computational studies should investigate the boundary signaling in light of these simulations. The teaching signals between reservoirs in the utilized model drove the populations towards consensus (similar TOPAs) when κ = 1 for both populations, rather than driving the populations towards opposite ends of the chunks. Further work can explore additional mechanisms on separating TOPAs for start/stop signaling. Our analysis was based on network and single cell dynamical constraints from animal (mostly rodent) data since human results are still limited, even though animal properties are not always directly translatable to human physiology. Both OCD and schizophrenia are heterogenous diseases. We sought to dissect the possible striatal $D_{1}$ and $D_{2}$ MSN and small network effects on sequence processing and did not consider possible larger circuit pathologies.

## Conclusion

To summarize, this study provides a mechanistic computational framework for some aspects of the $D_{1}$ and $D_{2}$ receptor-mediated chunk learning. In our model, the dopamine related modulation can contribute to chunk boundary signaling. Our modeling results imply that dynamical differences between the two segregated dopaminergic striatal populations may be advantageous, providing complementary functions for sequence start/stop recognition and execution. Some aspects of the functional dichotomy can likely be better explained by larger network modulations. We related our computational predictions to possible underlying neuropathophysiologies of OCD and schizophrenia symptoms (Szalisznyó and Silverstein 2019). Such biologically grounded approaches and diagnostics will contribute to the development of better informed dimensional taxonomies and classification systems of psychopathology (Sharma and Acharya 2021; Szalisznyó and Silverstein 2021).

## Data Availability

The datasets generated and analysed during the current study are available from the corresponding author on reasonable request.
